# Menstrual Statistics Based on a Study of 4,500 Menstrual Histories

**Published:** 1915-11

**Authors:** 


**Menstrual Statistics Based on a Study of 4,500 Menstrual Histories.** Dr. K. I. Sanes of Pittsburgh stated that 75 per cent of women menstruated regularly and 25 per cent irregularly. The most common regular type met with was that of 28 days, which constituted 72 per cent of this type. The 30 day type followed next in frequency with only 3.8 per cent, and the 21 day type with 3.3 per cent, etc. In some of the 31 days' type the menstrual flow appeared monthly on the same date independent of the number of days in the month. The most common irregular types were three to four weeks, then four to five weeks, two to three weeks, five to six weeks, etc. The most common ages of onset in order of their frequency were 15, 12, 16. The age of onset did not show any relation to regularity or irregularity of menstruation. The earliest age of onset in our series was 9 years and the latest 24 years. The most common duration of menstrual flow was three days, then four to five days, three to four days, five days, seven days, and four days. The irregular type of menstruation showed a larger percentage of long durations and smaller percentage of short durations, than the regular type. The quantity of flow in 45 per cent of women was normal, in 17 per cent scant, and in 37 per cent profuse. Irregular patients showed a higher percentage of profuse flow than the regular ones. Hyperthyroidism cases showed a very high percentage of profuse menstrual flow (65 per cent). Clots were very frequently found in menstrual flow and they did not find them to be influenced by menstrual irregularity. Forty-seven and four-tenths per cent of women suffered from dysmenorrhea (if the term dysmenorrhea was to convey the idea of discomfort). In retrodisplacements the percentage was found to be 50.4 per cent. In 50 per cent of the dysmenorrhea cases the symptoms appeared during the flow, in 29 per cent before, in 17 per cent before and after, and in the rest after the flow or during and after. Most frequently the dysmenorrhea appeared the day before the flow, then the first day of the flow, then two days during, two days before, one day before and two days during, then three days before, and seven days before. Improvement in menstruation was frequently noticed with advance of menstrual life, after marriage and after childbirths, menstrual periods becoming more regular and the dysmenorrhea improving or disappearing entirely. The most common ages of menopause in order of their frequency were given as 50, 46, 48, 47, 51, 49, 44, 45, 52. The length of menstrual life was commonly given as 37 years, then 35 and 33. Sixty-eight per cent reported a menstrual life of 30 years or more. The very early and very late ages of puberty showed a rather early menopause.— Am. Asso. Obs and Gyn. in Med. Rec.

